# Clinical score to predict the risk of bile leakage after liver resection

**DOI:** 10.1186/s12893-016-0147-0

**Published:** 2016-05-06

**Authors:** Takahiro Kajiwara, Yutaka Midorikawa, Shintaro Yamazaki, Tokio Higaki, Hisashi Nakayama, Masamichi Moriguchi, Shingo Tsuji, Tadatoshi Takayama

**Affiliations:** Department of Digestive Surgery, Nihon University School of Medicine, 30-1 Oyaguchikami-cho, Itabashi-ku, Tokyo, 173-8610 Japan; Genome Science Division, Research Center for Advanced Science and Technologies, University of Tokyo, 4-6-1 Komaba, Meguro-ku, Tokyo, 153-8904 Japan

**Keywords:** Postoperative complication, Bile leakage, Risk score

## Abstract

**Background:**

In liver resection, bile leakage remains the most common cause of operative morbidity. In order to predict the risk of this complication on the basis of various factors, we developed a clinical score system to predict the potential risk of bile leakage after liver resection.

**Methods:**

We analyzed the postoperative course in 518 patients who underwent liver resection for malignancy to identify independent predictors of bile leakage, which was defined as “a drain fluid bilirubin concentration at least three times the serum bilirubin concentration on or after postoperative day 3,” as proposed by the International Study Group of Liver Surgery. To confirm the robustness of the risk score system for bile leakage, we analyzed the independent series of 289 patients undergoing liver resection for malignancy.

**Results:**

Among 81 (15.6 %) patients with bile leakage, 76 had grade A bile leakage, and five had grade C leakage and underwent reoperation. The median postoperative hospital stay was significantly longer in patients with bile leakage (median, 14 days; range, 8 to 34) than in those without bile leakage (11 days; 5 to 62; *P* = 0.001). There was no hepatic insufficiency or in-hospital death. The risk score model was based on the four independent predictors of postoperative bile leakage: non-anatomical resection (odds ratio, 3.16; 95 % confidence interval [CI], 1.72 to 6.07; *P* < 0.001), indocyanine green clearance rate (2.43; 1.32 to 7.76; *P* = 0.004), albumin level (2.29; 1.23 to 4.22; *P* = 0.01), and weight of resected specimen (1.97; 1.11 to 3.51; *P* = 0.02). When this risk score system was used to assign patients to low-, middle-, and high-risk groups, the frequency of bile leakage in the high-risk group was 2.64 (95 % CI, 1.12 to 6.41; *P* = 0.04) than that in the low-risk group. Among the independent series for validation, 4 (5.7 %), 16 (10.0 %), and 10 (16.6 %) patients in low-, middle, and high-risk groups were given a diagnosis of bile leakage after operation, respectively (*P* = 0.144).

**Conclusions:**

Our risk score model can be used to predict the risk of bile leakage after liver resection.

## Background

Liver resection is widely accepted as the only potentially curative treatment for primary or metastatic liver malignancy. In high-volume centers, the mortality rate associated with liver resection has decreased [1–3]. However, the incidence of bile leakage, one of the most common complications after liver resection, remains high, ranging from 3.6 to 12.9 % [3–12].

Many different definitions of bile leakage have been proposed [3–12], most of which were based on both the bilirubin concentration and the amount of drain fluid. We previously defined bile leakage as continuous drainage with a bilirubin level of 5 mg/dl or higher beyond 1 week [13]. A bilirubin concentration of 20 mg/dl in drain fluid persisting for 2 weeks has also been proposed [5]. On the basis of drain fluid volume, bile leakage has been defined as the drainage of ≥50 ml of bile for longer than 1 to 3 days [8, 14]. On the other hand, some authors have defined bile leakage as the intra-abdominal accumulation of bile confirmed at reoperation or on percutaneous drainage or as the presence of cholangiographic evidence of biliary leakage. Finally, a uniform definition (“a drain discharge with a bilirubin concentration 3 times the serum level on after postoperative day 3”) and grading of bile leakage were established by the International Study Group of Liver Surgery (ISGLS) [15].

Despite the use of various procedures to decrease the risk of bile leakage, such as bile leakage tests [13], intraoperative cholangiography [16], and application of fibrin glue to the cut surface of the liver [17], this complication is not completely avoidable. Most cases of minor bile leakage are controllable and can be cured by conservative treatments [11]. On the other hand, the management of bile leakage is often difficult in patients with refractory ascites followed by the development of intra-abdominal sepsis after liver resection, resulting in a prolonged hospital stay or operative death [18]. Major bile leakage can lead to intractable ascites and liver failure unless it is treated appropriately. It is therefore clinically important to identify patients at high risk for bile leakage.

Despite the high incidence of bile leakage after liver resection, how to accurately assess the risk of this complication remains unclear. We therefore developed a risk score system for bile leakage on the basis of risk factors after liver resection.

## Methods

### Patients

Between 2008 and 2010, we performed curative liver resection without biliary reconstruction in 518 consecutive patients; this study received an approval (protocol number: RK-101208-6) by the institutional review boards of Nihon University, and each participant provided written informed consent. Clinical investigations were conducted according to the principles expressed in the Declaration of Helsinki. All the surgical procedures were performed via laparotomy. The diagnosis was hepatocellular carcinoma (HCC) in 364 patients, metastatic liver cancer in 130, intrahepatic cholangiocarcinoma in 14, liver invasion by an extrahepatic tumor in 2, malignant lymphoma in 1, and benign liver tumor in 7. There were 364 men and 154 women, with a median age of 68 years (range, 20 to 84).

To confirm the robustness of the risk score system for bile leakage (described below in detail), we analyzed the independent series of 289 patients undergoing liver resection for malignancy between 2011 and 2012.

### Surgical procedures

The indications for surgical resection and the operative procedures were determined in accordance with Makuuchi’s criteria [19]. Anatomic resection of Couinaud’s segment was the first-line operative procedure for HCC. Non-anatomic resection was performed in patients with colorectal metastases. Minor liver resection was defined as limited resection or resection of up to two Couinaud’s segments, and major liver resection was defined as resection of more than two segments. Hepatic parenchymal transection was guided ultrasonographically and performed by the clamp-crushing method with the inflow-blood-occlusion technique. Glisson’s pedicles were tied with silk thread and divided [13, 20].

At the end of the resection procedure, bile leakage was checked by placing a piece of gauze on the transected surface of the liver to confirm the presence or absence of bile staining. In patients who underwent resection of multiple segments of the liver or hemi-hepatectomy, bile leakage tests were routinely performed through the cystic duct of the gallbladder [13]. A fibrin glue preparation (Beriplast®; CSL Bering, Ltd., Tokyo, Japan) was applied to the raw surface of the liver.

A silicone rubber, closed irrigation drain (inner diameter, 10 mm) with one hole at the tip and two side holes (Pleats drain®; Sumitomo Co., Ltd., Tokyo, Japan) was placed in each cut surface of the liver via the shortest route from the abdominal wall. Standard systemic antibiotic therapy with cefazolin was routinely administered immediately before surgery and then twice daily on postoperative day (POD) 1 to 3. The drainage tube was managed as described previously [21]. Briefly, the drains were removed on POD 3 if the drainage fluid bilirubin level was less than 5 mg/dl and bacteriological cultures were negative.

Two consultant surgeons (T.T. and T.H.) performed three quarters of the operations. The first had done 3,000 liver resections, and the second had performed 1,000. Five resident specialists did a quarter of the operations while being assisted by the consultant. Neither the surgical nor anesthetic technique was modified during the study period.

### Definition of bile leakage

Bile leakage after liver resection was defined according to the criteria proposed by ISGLS [22]. Briefly, bile leakage was defined as a discharge of fluid with an increased bilirubin concentration via the intra-abdominal drains on or after POD 3 or as the need for radiologic intervention and relaparotomy for bile collection and bile peritonitis, respectively. An increased bilirubin concentration in the drain fluid was defined as a bilirubin concentration at least 3 times higher than the serum bilirubin concentration at the same time. Bile leakage was graded according to the system proposed by the ISGLS [15]. Grade A bile leakage has little or no impact on patients’ clinical management, and additional diagnostic or therapeutic interventions are unnecessary. A bile leakage that requires a change in patients’ clinical management, but can be treated without relaparotomy is defined as Grade B. A Grade A bile leakage requiring drainage for more than 1 week is also classified as Grade B. Patients with Grade C bile leakage require relaparotomy to control this complication.

Reoperation was performed when the amount of a discharge of fluid with a bilirubin concentration more than 50 mg/dl via drains was more than 50 ml/day.

### Risk score

Risk factors for bile leakage were defined as follows: clinicopathological factors that were associated with bile leakage on univariate analyses were included in logistic regression analysis (Table 1). Independent risk factors for bile leakage identified by the logistic regression model were weighted according to the odds ratios for postoperative bile leakage, and the point scores for variables related to bile leakage were calculated. Each patient was then assigned a total score.

**Table 1:** Bile leakage

	Healed spontaneously	Reoperation	*P* value
	(*N* = 76)	(*N* = 5)^a^	
Bilirubin concentration, mg/dl (range)	3.0 (1.4–42.1)	26.9 (5.3–55.2)	<0.001
Culture, n (%)	2 (2.6 %)	2 (40 %)	0.092
Discharge, day (range)	14 (8–28)	24 (13–34)	0.007
Drain removal	8 (4–191)	40 (14–107)	0.029

^a^one patient underwent both percutaneous drainage and reoperation

### Statistical analysis

Comparisons between variables collected from the patients who underwent liver resection were evaluated with Fisher’s exact test for categorical variables and the Welch two sample *t*-test for ratios.

The durations of abdominal drainage and the hospital stay were calculated with the Kaplan-Meier method and were compared using the log-rank test. *P* values of less than 0.05 were considered to indicate statistical significance. All analyses were performed using a statistical software package (JMP version 8.0, SAS, NC).

## Results

### Bile leakage

Among the 518 patients who underwent liver resection for malignancy, bile leakage was diagnosed in 81 (15.6 %). Seventy-six of these patients did not require radiologic intervention or relaparotomy (94 %), and all of their drainage tubes were removed within 1 week (Grade A). The other five patients underwent relaparotomy to treat bile leakage (Grade C, 6 %) (Table 1).

The median time to removal of the drain after operation was significantly longer in patients with bile leakage (9 days; range, 8 to 34) than in those without bile leakage (5 days; 5 to 62, *P* < 0.001). The median day of discharge was POD 24 (range, 13 to 34) in the 5 patients who underwent reoperation, as compared with POD 14 (range, 8 to 28) in the 76 patients who did not undergo relaparotomy (*P* < 0.001).

### Postoperative course

**Fig. 1:**
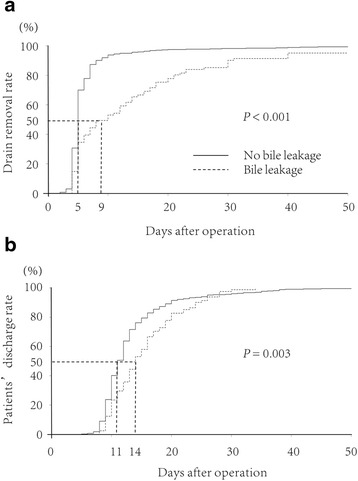
Cumulative rates of the postoperative drain removal (**a**) and patients’ discharge (**b**). There were significant differences between the two groups in both the duration of abdominal drainage (*P* < 0.001) and the discharge rate (*P* = 0.003)

The median day of drain removal was POD 5 (range, 2 to 65) in the patients without bile leakage and POD 10 (range, 4 to 191) in the patients with bile leakage (*P* < 0.001) (Fig. 1a). The median postoperative hospital stay was 11 days (range, 5 to 62) in the patients without bile leakage and 14 days (range, 8 to 34) in those with bile leakage (*P* = 0.003) (Fig. 1b). No patient had hepatic failure, and there was no operative or in-hospital death.

### Risk factors

Among the 18 clinicopathological factors studied, indocyanine green clearance rate at 15 min (ICGR15) (*P* = 0.02), albumin level (*P* = 0.04), operation time (*P* < 0.001), ischemia time (*P* = 0.008), blood loss (*P* = 0.004), anatomical resection (*P* = 0.01), vascular invasion (*P* = 0.04), and weight of resected specimen (*P* = 0.006) were significantly related to bile leakage (Table 2).

**Table 2:** Univariate analyses

	Bile leakage	Control	*P* value
	(*n* = 81)	(*n* = 437)	
Age, years (range)	68 (44–84)	68 (20–84)	0.91
Background liver, NL/CH + LC	26/55	143/294	0.91
ICGR15, % (range)	9.95 (2.05–34.83)	11.38 (1.14–43.1)	0.03*
Child-Pugh, A/B	80/1	431/6	0.92
Diabetes mellitus (+/−)	22/59	118/319	0.92
Steatosis, +/−	31/50	137/300	0.29
Aspartate transamonase, IU/L (range)	28 (9–213)	32 (11–265)	0.06
Total bilirubin, μmol/L (range)	0.58 (0.19–1.62)	0.59 (0.19–3.51)	0.37
Platelet count, ×10^5 (range)^	17.9 (5–41.4)	16.4 (4.2–54.9)	0.26
Albumin, g/L (range)	3.8 (2.7–4.7)	4.1 (2.4–5.3)	0.04*
Operation time, min (range)	425 (141–752)	356 (115–803)	0.004*
Ischemia time, min (range)	91 (10–240)	72 (0–243)	0.008*
Intraoperative blood loss, ml (range)	407 (17–3777)	266 (10–850)	0.004*
Anatomic resection, +/−	16/65	144/293	0.01*
Primary/repeated	63/18	108/329	0.64
Resected number, single/multiple	59/22	321/116	0.9
Vascular invasion, +/−	20/61	62/375	0.04*
Weight of resected specimen, g	90 (10–730)	64.5 (2–1635)	0.006*

*significant difference between 2 groups

*NL* normal liver, *CH* chronic hepatitis, *LC* liver cirrhosis, *ICGR15* indocyanine green clearance rate at 15 min, *AST* aspartate aminotransferase, *HCC* hepatocellular carcinoma

On multivariate analysis (Table 3), the independent factors for bile leakage were non-anatomical resection (odds ratio [OR], 3.16; 95 % confidence interval [CI], 1.72 to 6.07; *P* < 0.001), ICGR15 (2.43; 1.32 to 7.76; *P* = 0.004), albumin level (2.29; 1.23 to 4.22; *P* = 0.01), and weight of resected specimen (1.97; 1.11 to 3.51; *P* = 0.02).

**Table 3:** Multivariate analyses

	Odds ratio (95 % CI)	Score	*P* value
Non-anatomical resection	3.16 (1.72–6.07)	2	0.0001
ICGR15 (<15 %)	2.43 (1.32–7.76)	1	0.004
Albumin (g/L) (3.5≧)	2.29 (1.23–4.22)	1	0.01
Weight of resected specimen	1.97 (1.11–3.51)	1	0.02

95 % CI, 95 % confidence interval

*ICGR15* indocyanine green clarance rate at 15 min

### Risk score system

In the risk score system, each of the following factors was assigned a score of 1 point: ICGR15 less than 15 %, serum albumin level less than 3.5 g/L, and weight of resected specimen less than 70 g. If non-anatomical resection was performed, two points were assigned because the odds ratio of this factor was much higher than that of the other risk factors (Table 3).

Patients with a risk score of 1 or less were assigned to the low-risk group, those with a risk score of 2 or 3 to the middle-risk group, and those with a risk score 4 or higher to the high-risk group (Table 4). Among the 122 (23.5 %) patients in the low-risk group, 316 (61.0 %) in the middle-risk group, and 80 (15.5 %) in the high-risk group, bile leakage was diagnosed after liver resection in 12 (9.8 %), 51 (16.1 %), and 18 (22.5 %) patients, respectively (*P* = 0.04).

**Table 4:** Risk score for bile leakage

Risk score	No. of patients (%)	Bile leakage (%)	Odds ratio (95 % CI)^a^	*P* value
Low	122 (23.5)	12 (9.8)	1	0.04
Middle	316 (61.0)	51 (16.1)	1.76 (0.88–3.77)	
High	80 (15.5)	18 (22.5)	2.64 (1.12–6.45)	

Low risk, risk score 0 or 1; Middle risk, 2 or 3; High risk, 4 or 5, respectively

^a^Each odds ratio was calculated relative to the low-risk group

The independent series for validation contained 69 (23.8 %) patients in the low-risk group, 160 (55.3 %) in the middle-risk group, and 60 (20.7 %) in the high-risk group, and 4 (5.7 %), 16 (10.0 %), and 10 (16.6 %) patients were given a diagnosis of bile leakage after operation, respectively (*P* = 0.144). Although there were no significant difference between each group, the patients in the high-risk group tended to suffer from bile leakage in the independent series of 289 patients.

## Discussion

In this study, we developed a risk score to identify patients at high risk for bile leakage after liver resection. Our system was based on four independent factors (non-anatomical resection, shorter ICGR15, low albumin level, and weight of resected specimen) in 518 patients undergoing liver resection that were found to be independently related to the risk of bile leakage, and was confirmed using the another series of 289 patients who underwent liver resection in the next 2 years.

Because a range of variables contribute to prediction models, empirical weights are necessary. Regression models generated by a mathematical approach are usually used to classify patients according to the risk of predefined events [23]. However, the complexity of regression models can make them unsuitable for clinical use [24]. In this study, we identified variables related to the risk of bile leakage and assigned these variables empirical weights in accordance with the odds ratios. This process simplified the regression model to facilitate routine clinical use. With the use of our scoring system, the patients were clearly divided into three risk groups for bile leakage.

We determined the extent of liver resection according to the Makuuchi criteria [19], which is based on only ICGR15, serum total bilirubin level, encephalopathy, and ascites. Therefore, a patient with a lower ICGR15 and serum albumin level could be a candidate for more extensive liver resection. In conjunction with weight of resected specimen more than 70 g [21], large resection of the liver for relatively poorer liver function could harbors high risk for bile leakage by the score system.

In this study, anatomical resection decreased the risk of bile leakage. This is because, in anatomical resection, Glisson’s sheath is ligated at the central side, and the number of peripheral branches appearing in the cut surface is theoretically small [22]. Exposure of major Glisson’s sheath is also rare, which was reported to be one of independent risk factors for bile leakage [7, 8, 25].

It has been reported that the use of a drainage tube is no longer necessary in patients who undergo liver resection [26, 27]. However, our results and those of previous studies have shown that bile leakage after liver resection is not a rare event, occurring in about 20 % of patients, and that high-risk patients can be identified by means of a clinical risk score. We therefore advocate that a drainage tube should be placed, especially in high-risk patients.

As for the management of bile leakage, Vigano et al. reported that conservative management was successful in 76 % of patients and that a drainage output of greater than 100 ml on POD 10 was a predictor of conservative management failure [11]. However, even patients in whom leakage spontaneously resolved had a median waiting time of 15 days, and the hospital stay in patients with no response to conservative treatment was prolonged. In our series, 5 of the 81 patients with bile leakage underwent reoperation. The median postoperative hospital stay in patients who underwent reoperation was 24 days, as compared with 14 days in patients who did not undergo reoperation. Given the results of a previous study of bile leakage treated conservatively [11], the postoperative hospital stay in patients who received reoperation was quite short. We therefore assume that patients who have major leakage that shows no improvement on POD 3 are good candidates for reoperation [11, 28].

Drainage after liver resection is controversial by recent randomized controlled trials [26, 27, 29]. However, our results show that there definitely exists a subgroup of patients at high risk for bile leakage and that this risk can be preoperatively predicted by the risk score system. Especially, bile leakage was observed within three postoperative days, and after drain removal, bile leakage in which treatments had been required did not occur. Therefore, we emphasize the need for drainage after liver resection, especially in high-risk patients, as well as early removal of prophylactic drains according to appropriate criteria and the risk score [21].

## Conclusion

Our risk score system, simply based on four clinical factors, effectively predicted the risk of bile leakage after liver resection. Patients with a high-risk score thus require more meticulous management by an expert surgeon and the use of standardized techniques to avoid this complication.

## Ethics approval and consent to participate

This study received an approval (protocol number: RK-101208-6) by the institutional review boards of Nihon University, and each participant provided written informed consent.

## Availability of data and materials

The dataset supporting the conclusions of this article is included within the article.

## Abbreviations

HCC: hepatocellular carcinoma
ICGR15: indocyanine green clearance rate at 15 min
ISGLS: the International Study Group of Liver Surgery
POD: postoperative day

## References

[1] Imamura H, Seyama Y, Kokudo N, Maema A, Sugawara Y, Sano K (2003). One thousand fifty-six hepatectomies without mortality in 8 years. Arch Surg.

[2] Makuuchi M, Sano K (2004). The surgical approach to HCC: our progress and results in Japan. Liver Transpl.

[3] Takayama T (2011). Surgical treatment for hepatocellular carcinoma. Jpn J Clin Oncol.

[4] Benzoni E, Cojutti A, Lorenzin D, Adani GL, Baccarani U, Favero A (2007). Liver resective surgery: a multivariate analysis of postoperative outcome and complication. Langenbecks Arch Surg.

[5] Capussotti L, Ferrero A, Viganò L, Sgotto E, Muratore A, Polastri R (2006). Bile leakage and liver resection: Where is the risk?. Arch Surg.

[6] Kyoden Y, Imamura H, Sano K, Beck Y, Sugawara Y, Kokudo N (2010). Value of prophylactic abdominal drainage in 1269 consecutive cases of elective liver resection. J Hepatobiliary Pancreat Sci.

[7] Lee CC, Chau GY, Lui WY, Tsay SH, King KL, Loong CC (2005). Risk factors associated with bile leakage after hepatic resection for hepatocellular carcinoma. Hepatogastroenterology.

[8] Lo CM, Fan ST, Liu CL, Lai EC, Wong J (1998). Biliary complications after hepatic resection: risk factors, management, and outcome. Arch Surg.

[9] Poon RT, Fan ST, Lo CM, Liu CL, Lam CM, Yuen WK (2004). Improving perioperative outcome expands the role of hepatectomy in management of benign and malignant hepatobiliary diseases: analysis of 1222 consecutive patients from a prospective database. Ann Surg.

[10] Tanaka S, Hirohashi K, Tanaka H, Shuto T, Lee SH, Kubo S (2002). Incidence and management of bile leakage after hepatic resection for malignant hepatic tumors. J Am Coll Surg.

[11] Viganò L, Ferrero A, Sgotto E, Tesoriere RL, Calgaro M, Capussotti L (2008). Bile leak after hepatectomy: predictive factors of spontaneous healing. Am J Surg.

[12] Virani S, Michaelson JS, Hutter MM, Lancaster RT, Warshaw AL, Henderson WG (2007). Morbidity and mortality after liver resection: results of the patient safety in surgery study. J Am Coll Surg.

[13] Ijichi M, Takayama T, Toyoda H, Sano K, Kubota K, Makuuchi M (2000). Randomized trial of the usefulness of a bile leakage test during hepatic resection. Arch Surg.

[14] de Castro SM, Kuhlmann KF, Busch OR, van Delden OM, Laméris JS, van Gulik TM (2005). Incidence and management of biliary leakage after hepaticojejunostomy. J Gastrointest Surg.

[15] Koch M, Garden OJ, Padbury R, Rahbari NN, Adam R, Capussotti L (2011). Bile leakage after hepatobiliary and pancreatic surgery: a definition and grading of severity by the International Study Group of Liver Surgery. Surgery.

[16] Kubo S, Sakai K, Kinoshita H, Hirohashi K (1986). Intraoperative cholangiography using a balloon catheter in liver surgery. World J Surg.

[17] Kohno H, Nagasue N, Chang YC, Taniura H, Yamanoi A, Nakamura T (1992). Comparison of topical hemostatic agents in elective hepatic resection: a clinical prospective randomized trial. World J Surg.

[18] Yamashita Y, Hamatsu T, Rikimaru T, Tanaka S, Shirabe K, Shimada M (2001). Bile leakage after hepatic resection. Ann Surg.

[19] Makuuchi M, Kosuge T, Takayama T, Yamazaki S, Kakazu T, Miyagawa S (1993). Surgery for small liver cancers. Semin Surg Oncol.

[20] Imamura H, Takayama T, Sugawara Y, Kokudo N, Aoki T, Kaneko J (2002). Pringle's manoeuvre in living donors. Lancet.

[21] Yamazaki S, Takayama T, Moriguchi M, Mitsuka Y, Okada S, Midorikawa Y (2012). Criteria for drain removal following liver resection. Br J Surg.

[22] Takayama T, Makuuchi M, Kubota K, Harihara Y, Hui AM, Sano K (2001). Randomized comparison of ultrasonic vs clamp transection of the liver. Arch Surg.

[23] Freedman DA. Statistical Models: Theory and Practice. New York: Cambridge University Press. 2009;41-60.

[24] Infante-Rivard C, Esnaola S, Villeneuve JP (1987). Clinical and statistical validity of conventional prognostic factors in predicting short-term survival among cirrhotics. Hepatology.

[25] Yoshioka R, Saiura A, Koga R, Arita J, Takemura N, Ono Y (2011). Predictive factors for bile leakage after hepatectomy: analysis of 505 consecutive patients. World J Surg.

[26] Belghiti J, Kabbej M, Sauvanet A, Vilgrain V, Panis Y, Fekete F (1993). Drainage after elective hepatic resection. A randomized trial. Ann Surg.

[27] Liu CL, Fan ST, Lo CM, Wong Y, Ng IO, Lam CM (2004). Abdominal drainage after hepatic resection is contraindicated in patients with chronic liver diseases. Ann Surg.

[28] Ishii H, Ochiai T, Murayama Y, Komatsu S, Shiozaki A, Kuriu Y (2011). Risk factors and management of postoperative bile leakage after hepatectomy without bilioenteric anastomosis. Dig Surg.

[29] Fong Y, Brennan MF, Brown K, Heffernan N, Blumgart LH (1996). Drainage is unnecessary after elective liver resection. Am J Surg.

